# Increasing Opportunities for Question-Asking in School-Aged Children with Autism Spectrum Disorder: Effectiveness of Staff Training in Pivotal Response Treatment

**DOI:** 10.1007/s10803-016-2966-3

**Published:** 2016-11-25

**Authors:** Rianne Verschuur, Bibi Huskens, Ludo Verhoeven, Robert Didden

**Affiliations:** 1 Behavioural Science Institute, Radboud University, Montessorilaan 3, 6525 HR Nijmegen, The Netherlands; 2 Dr. Leo Kannerhuis, Department of Research, Development & Innovation, Houtsniplaan 1, 6568 ZH Doorwerth, The Netherlands; 3 Behavioural Science Institute, Radboud University, P.O. Box 9104, 6500 HE Nijmegen, The Netherlands

**Keywords:** Autism spectrum disorder, Pivotal response treatment, Staff training, Question-asking

## Abstract

Deficits in question-asking are common in children with autism spectrum disorder (ASD). Furthermore, their opportunities to self-initiate questions are often hindered by directive behavior of their conversation partners. This study assessed the effectiveness of staff training in pivotal response treatment (PRT) on staff member-created opportunities and self-initiated questions of school-aged children with ASD. Generalization and maintenance were also assessed. Participants were 14 staff members and children with ASD attending an inpatient treatment facility. Data showed that PRT resulted in significant increases in both staff member-created opportunities and child-initiated questions. Generalization to group situations and collateral changes in children’s language, pragmatic, and adaptive skills, and maladaptive behaviors did not occur. Implications for clinical practice and directions for future research are discussed.

## Introduction

Autism spectrum disorder (ASD) is characterized by restricted, repetitive, and stereotyped behaviors and impairments in social communication and social interaction, (American Psychiatric Association 2013), including deficits in self-initiations and question-asking. Compared with typically developing children, children with ASD ask fewer questions and their questions serve fewer functions (Hauck et al. 1995; Stone and Caro-Martinez 1990; Stone et al. 1997; Wetherby and Prutting 1984). This results in reduced opportunities for learning a variety of skills as they elicit fewer teaching interactions from their environment (Koegel et al. 2003; McDuff et al. 2001). Furthermore, deficits in question-asking often lead to directive behavior of children’s environment, thereby further reducing their opportunities to self-initiate questions (Hudry et al. 2013). Deficits in question-asking are associated with poorer long-term outcomes on pragmatic and adaptive skills and school and community functioning (Koegel et al. 1999). For these and other reasons, it is important to teach children with ASD to initiate questions.

Numerous studies have reported on interventions aimed at teaching question-asking skills to children with ASD. Targeted questions had various communicative functions, including requesting objects (e.g., Wert and Neisworth 2003), help (e.g., Dotto-Fojut et al. 2011), information (e.g., Betz et al. 2010), and social information (e.g., Dogget et al. 2013). These studies encompassed multicomponent behavioral interventions to increase question-asking, for example discrete trial teaching (DTT; e.g., Ingvarsson and Hollobaugh 2010), pivotal response treatment (PRT) (e.g., Koegel et al. 2014), self-management (e.g., Koegel et al. 2014), and video modeling (e.g., Charlop and Millstein 1989). Common components included contrived establishing operations, systematic prompting (e.g., echoic prompts) and prompt fading procedures (e.g., time delay) and natural reinforcement (Raulston et al. 2013). A systematic review reported positive results of these components with regard to the acquisition of targeted questions, suggesting that these components are effective in teaching question-asking skills to children with ASD (Raulston et al. 2013). However, the effectiveness of these components has not yet been investigated during intervention sessions in the context of natural everyday activities, conducted by children’s natural conversational partners, and targeting questions with various communicative functions. Moreover, generalization effects of question-asking interventions to natural situations were rather limited (e.g., Betz et al. 2010). Deficits in question-asking in natural situations may thus reflect a performance deficit rather than a skill deficit (Koegel et al. 2012, 2014; Palmen et al. 2008). To bring question-asking under control of natural stimuli, children with ASD should preferably be taught question-asking skills directly in natural situations by natural conversational partners who need training to implement interventions with adequate treatment fidelity (e.g., Reid and Fitch 2011).

Pivotal response treatment may be indicated, because training in the child’s natural environment is a critical component of PRT (Koegel and Koegel 2006). PRT is an intervention model derived from the principles of applied behavior analysis (ABA) that targets pivotal skills (e.g., self-initiations) in children with ASD in order to achieve generalized improvements in their functioning. A systematic review found evidence for the effectiveness of PRT for increasing self-initiations including question-asking in children with ASD (see Verschuur et al. 2014). Furthermore, evidence for generalized improvements in language, communication, play, affect and maladaptive behavior as a result of PRT was reported. However, studies on the effectiveness of PRT on question-asking skills have several limitations. First, although training children in their natural environment is a key component of PRT, in studies where PRT was implemented to improve question-asking skills, PRT sessions were usually not conducted during natural everyday activities (e.g., Koegel et al. 2010) and PRT was not implemented by children’s natural conversation partners (e.g., Doggett et al. 2013). Second, the effectiveness of PRT has mainly been investigated in preschool children with ASD (e.g., Koegel et al. 2003, 2014). The few studies that investigated the effectiveness of PRT on self-initiations (including asking questions) in school-aged children with ASD reported either positive results (e.g., Dogett et al. 2013; Robinson 2011) or mixed results (e.g., Huskens et al. 2012). They also failed to measure gains in collateral skills. The latter is important because PRT assumes that collateral skills improve as a result of the acquisition of pivotal skills. Third, the effectiveness of PRT on question-asking has not yet been investigated in children with ASD receiving inpatient treatment. This may be viewed as a limitation as approximately 6% of children with ASD receive inpatient treatment (e.g., Cidav et al. 2013), predominantly because of psychiatric comorbidity, aggressive behavior, self-injurious behavior, and impaired emotion regulation (Mandell 2008; Siegel and Gabriels 2014). It is unclear whether PRT is effective for school-aged children who are admitted to an inpatient facility and whether their staff is able to implement PRT in daily one-to-one situations.

This study aimed to investigate (a) effectiveness of PRT staff training on staff member-created opportunities, (b) effectiveness of PRT on self-initiated questions of school-aged children with ASD during everyday activities in one-to-one situations, (c) generalization of these skills to group situations, and (d) maintenance of these skills over a 6-month period. Furthermore, collateral changes in children’s language, pragmatic, and adaptive skills and maladaptive behaviors were explored.

## Method

### Setting and Participants

The study was conducted at an inpatient treatment facility for children with ASD in the Netherlands. Fourteen staff members (13 females) and 14 children (13 males) with ASD participated. Staff members had a mean age of 30 years (range 23–42) at baseline. The highest level of education was secondary school for one staff member; 13 staff members had a bachelor’s degree. On average, they had 5:8 years of experience with children with ASD (range 1:5–13:5), worked at the facility for 4:3 years (range 0:4–9:3), and for 27.5 h per week (range 24–32). They had no experience with PRT prior to this study. Children were included if they met the following criteria: (a) diagnosis of ASD according to the DSM-IV-TR criteria (American Psychiatric Association 2000), confirmed by scores on the Social Communication Questionnaire [(SCQ), Rutter et al. 2004] and/or Autism Diagnostic Observation Schedule [(ADOS-2), Lord et al. 2012; Dutch version by De Bildt et al. 2013], (b) aged between 6 and 14 years at baseline, (c) total IQ or verbal and performance IQ above 70 on the Dutch version of the Wechsler Intelligence Scale for Children-III (WISC-III^NL^; Kort et al. 2005) or the Nederlandse Intelligentietest voor Onderwijs niveau (Dutch intelligence scale for educational level; Dijk and Tellegen 2004), (d) ability to communicate verbally, (e) median percentage of self-initiated questions below 50 during baseline (see “Child-Initiated Questions”), and (f) receiving inpatient treatment during the period of data collection, at least up to and including the post-intervention phase (see “Procedures”). Children received inpatient treatment because of severe autism symptoms, psychiatric comorbidity, maladaptive behaviors, or an exceeding of parents’ ability to cope with the demands of parenting a child with ASD. The purpose of inpatient treatment was to teach skills to children with ASD and their families and to reduce children’s maladaptive behaviors so that children could return to their families. The average duration of inpatient treatment was 1 year. Children were discharged if their inpatient treatment goals were met. Discharge from the inpatient treatment facility was not related to participation in the present study. Informed consent was obtained from the parents of each child. The study was approved by the Ethics Committee of the Faculty of Social Sciences of the Radboud University, Nijmegen, the Netherlands (ECG2013-1304-100).

Demographic characteristics of the children are displayed in Table 1. They had a mean age of 11:6 years at baseline (range 7:7–13:5) and their scores on the SCQ and ADOS-2 confirmed the ASD diagnosis.

**Table 1:** Demographic characteristics of children at baseline

Child	Age	Diagnosis	SCQ^a^	ADOS-2^b^	IQ
1	12:9	PDD-NOS, ADHD, tic disorder NOS	28	20	81 (V), 96 (P)
2	12:5	Autistic disorder, ADHD	26	16	79
3	11:9	Autistic disorder	19	11	97 (V), 78 (P)
4	13:4	Asperger’s disorder	23	15	126
5	7:7	Autistic disorder, ADHD	25	8	132
6	10:0	Autistic disorder	23	15	87 (V), 107 (P)
7	10:3	Autistic disorder, ADHD	22	12	87 (V), 109 (P)
8	11:5	PDD-NOS, reactive attachment disorder	26	15	88 (V), 105 (P)
9	9:5	Autistic disorder	18	15	69 (V), 101 (P)
10	13:5	PDD-NOS	21	9	94
11	13:5	Autistic disorder	18	20	128 (V), 91 (P)
12	11:2	Autistic disorder	27	10	89
13	12:1	PDD-NOS (subtype Multiple Complex Developmental Disorder), ADHD	26	9	90
14	12:2	Asperger’s disorder	18	9	114 (V), 92 (P)

Reported ages are in years:months

^a^Score >15 on the SCQ is an indication for ASD

^b^Reported scores are ADOS-2 total scores; a score ≥7 is an indication for ASD

### Design

A multiple baseline design across three groups of staff members and children was used to investigate the effectiveness of PRT staff training on staff-member created opportunities and child-initiated questions, and generalization and maintenance of these skills (Kazdin 2011). The facility consisted of three different treatment units. The three groups in the multiple baseline design corresponded with these three treatment units. To prevent interdependence of baselines, staff members and children were not randomly assigned to the groups (Kazdin 2011). To explore the effectiveness of PRT staff training on children’s language skills, pragmatic skills and adaptive skills pre-tests and post-tests were conducted.

### Procedures

#### Baseline

Baseline consisted of three to five sessions. Each staff member was paired with a child to form a dyad. The purpose of the baseline sessions was to assess whether staff members were creating opportunities for question-asking prior to participating in PRT staff training. The baseline sessions also served to assess the baseline level of child-initiated questions. Staff members were instructed to conduct 10-min one-to-one sessions with the child during age-appropriate everyday activities requiring interactions, such as playing a game, building with construction toys, drawing, and baking. If the child initiated a question during these activities, staff members were instructed to respond to the question as they usually did. Staff members and children completed the activities. Staff members received no feedback on their use of PRT techniques. They were asked to record baseline sessions using a video camera. Staff members were instructed to record a session (a) lasting at least 10 min, (b) recorded in a one-to-one situation, and (c) during which staff member, child and activity were visible and audible on camera. Next to this, staff members were instructed to fill in the Children’s Communication Checklist (CCC2) and Vineland-II parent/caregiver rating form about the child during the last 4 weeks of baseline (see “Measures of Collateral Changes” section).

#### Intervention

During intervention, staff members participated in a PRT staff training that was conducted by two licensed PRT supervisors. Both PRT supervisors were certified by the Koegel Autism Center and had more than 5 years experience in conducting PRT staff training. PRT staff training consisted of four 6-h sessions in which staff members were introduced to ABA and PRT. Furthermore, they received instruction in antecedent PRT techniques (i.e., incorporating the child’s choice, gaining the child’s attention, proving clear opportunities, and interspersing maintenance and acquisition tasks) and consequent PRT techniques (i.e., using contingent reinforcement, using natural reinforcement, and reinforcing attempts), discussed video-examples displaying the techniques, completed worksheets and took part in role-plays to practice techniques. They were also taught to set goals related to the pivotal behavior of self-initiations for the child in their dyad (i.e., requesting objects, help, information, or social information) and to record data on these goals.

After each session, staff members were asked to practice the PRT techniques during one-to-one PRT sessions and to videotape these sessions. During PRT sessions staff members and children first discussed the child’s goal (e.g., requesting help) after which an activity of the child’s choice was started. During the activity, staff members were required to create opportunities (i.e., trials) using PRT techniques to stimulate children to initiate questions (e.g., ‘Could you help me?’). During each trial, staff members first followed the child’s choice and gained his/her attention. If the child initiated a question or did a reasonable attempt, staff members reinforced this self-initiation contingently and naturally. If the child did not initiate a question within 5 s, staff members prompted the child to initiate. To increase the child’s motivation to self-initiate, staff members interspersed acquisition trials with maintenance trails. The PRT session ended when the activity was completed.

During session 2–4 of the PRT staff training, staff members received oral feedback from the PRT supervisors on their use of PRT techniques in 1-min fragments of the videotapes. They also received written feedback on their use of PRT techniques in 10-min fragments of the videotapes, including whether they had met the criterion for fidelity (i.e., 80%) of PRT implementation. To demonstrate fidelity of PRT implementation, staff members were required to implement each PRT technique during at least 80% of the intervals and to create at least one opportunity per minute (Koegel and Koegel 2006). The intervention phase continued until all staff members of the same group demonstrated fidelity of implementation in three 10-min videotapes with the child and two 10-min videotapes with two other children (i.e., to demonstrate generalization across children).

#### Post-intervention

Post-intervention consisted of three sessions. Procedures were similar to those during baseline. Staff members were instructed to conduct three 10-min one-to-one PRT sessions with the child during age-appropriate everyday activities and to videotape these sessions. If a child’s inpatient treatment was terminated before post-intervention started, staff members were paired with another child that already received PRT to conduct post-intervention sessions with. If staff members were not available during post-intervention (e.g., due to illness), children were paired with another staff member that was already participating in this study. This concerned one staff member and one child (7%). Staff members received no feedback on their use of PRT techniques. In addition, they were instructed to fill in the CCC2 and Vineland-II parent/caregiver rating form about the child (see “Measures of Collateral Changes” section) and to rate the social validity of the PRT staff training (see “Social Validity” section).

#### Follow-up

Follow-up data were collected during three sessions 6 months after the last post-intervention session. Because inpatient treatment was terminated for nine children and two staff members had left the facility, follow-up sessions were conducted for 12 staff members and five children. Staff members and children were paired again to form dyads. The procedures for follow-up sessions and requirements for videotapes were identical to those during post-intervention and baseline.

#### Generalization Probes

To assess generalization of staff members’ and children’s skills to group situations, generalization probes were conducted for five staff members and five children. During baseline and post-intervention three 10-min generalization probes were conducted for each staff member and each child during breakfast, lunch, afternoon tea, dinner, a group play situation inside and a group play situation outside in a random order. The researcher (i.e., first author) videotaped the generalization probes.

### Dependent Measures

#### Staff Member-Created Opportunities

An event-recording system was used to measure the number of staff member-created opportunities (Cooper et al. 2013). Ten minutes of the videotapes were viewed and scored by two observers naïve to the purpose of the study. When videotapes lasted more than 10 min, the 10 min in the middle of these videotapes were observed. Whereas PRT implementation is usually computed globally (i.e., dividing the number of minutes wherein all PRT techniques were implemented by the total number of minutes), the present study used the exact computation as proposed by Huskens et al. (2012). This exact computation assumes that a correct staff member-created opportunity consists of a sequence of correctly implemented PRT techniques. Two sequences were considered correct: (1) creating a clear opportunity, child-initiated question, and reinforcing the child’s question or attempt contingently and naturally, or (2) creating a clear opportunity, prompting the child to initiate a question, prompted question, and reinforcing the child’s question or attempt contingently and naturally. The following categories were recorded: (a) creating a clear opportunity, (b) child-initiated question, (c) prompting the child to initiate a question, (d) prompted question and (e) reinforcing the child’s question or attempt contingently and naturally. Operational definitions of the categories are presented in Table 2. An example of a correct and clear opportunity would be holding the dice during a game while it is the child’s turn and immediately giving the dice to child when he or she asked for it. If a staff member stated ‘I went to the zoo with my sister yesterday and it was fun’, the opportunity would be considered unclear, because the staff member’s statement included too much information and it was not clear which question the child could ask. Observers were instructed to record each sequence using numbers (i.e., 1 shared control, 2 child-initiated question, and 3 reinforcement). In order to determine inter-observer agreement observers were also instructed to record the point in time at which the staff member began to reinforce the child’s question or attempt (see “Inter-observer Agreement” section) For each staff member, the number of staff member-created opportunities was calculated by counting the number of correct sequences per 10-min videotape.

**Table 2:** Definitions of behavioral categories for opportunities

Behavioral category	Operational definition
Creating a clear opportunity	The staff member created a clear opportunity by (a) Shared control: the staff member had control over an object the child desired or needed during the activity (b) Visible, out of reach: the object the child desired or needed during the activity was visible, but out of the child’s reach; the object was neither in the staff member’s possession (c) Invisible, out of reach the object the child desired or needed during the activity was invisible and out of the child’s reach; the object was neither in the staff member’s possession (d) Waiting: the staff member did nothing and waited for 3 s, when the routine of the activity expected the staff member to act or when the child needed help to carry out an action (e) Breaking a routine: the staff member did something that did not fit in the routine of the activity, for example throwing the dice while it was the child’s turn (f) Interrupting: the staff member talked, but stopped in the middle of a sentence, for example ‘Now we need to add 100 grams of ...’ (g) Making a statement: the staff member made a statement or comment without giving details to the child, for example ‘I have done something fun yesterday’, and then remained silent
Child-initiated question	The child began or directed a social interaction by asking a question within 5 s after the member created a clear opportunity
Prompting the child to self-initiate	If the child did not initiate a question or did no reasonable attempt within 5 s after the staff member created a clear opportunity, the staff member offered help by prompting. Three types of prompts were recorded (a) Time delay prompt: the staff member was silent for three seconds, while giving the child a questioning look and/or making a sign to stimulate the child to respond (b) Open-ended question prompt: the staff member asked an open question to stimulate the child to initiate, for example ‘What could you ask me now?’ (c) Verbal model prompt: the staff member modeled the question or comment that the child could use to initiate The staff member continued prompting until the child initiated a question or did a reasonable attempt or until the staff member had used three prompts. The order of prompts was not predetermined
Prompted question	The child directed a social interaction by asking a question after the staff member prompted the child
Reinforcing the child’s question or attempt contingently and naturally	The staff member reinforced the child’s question or attempt naturally and contingently by responding to this initiation within 2 s. Contingent and natural reinforcement was only recorded if (a) the response was the staff member’s first behavior after the child’s initiation and (b) the response was a natural consequence of the child’s question (i.e. in everyday life the response to this question is equal)

#### Child-Initiated Questions

An interval-recording system was used to measure child-initiated questions (Cooper et al. 2013). Ten minutes of the videotapes were independently viewed and scored by two observers naïve to the purpose of this study. When videotapes lasted more than 10 min, the 10 min in the middle of these videotapes were observed. Videotapes were divided into 30 intervals of 20 s. The following categories were recorded per interval: (a) unprompted (i.e., spontaneous) correct question and (b) unprompted attempt to a question. A spontaneous child-initiated question was defined as the child asking a question that (a) began or directed a social interaction, (b) began with an interrogative (e.g., ‘Where...?’ or ‘With whom...?’) or verb (e.g., ‘May I...?’ or ‘Can you...?’) or had an interrogative intonation, and (c) was not directly preceded by a prompt. Questions that were part of an activity (e.g., ‘Does he have blond hair?’ in the game Who is it?) were not recorded. A child-initiated question was recorded as correct if the child directed the question to the staff member by orientating his/her face to the staff member or calling the staff member’s name. A child-initiated question was recorded as an attempt when the child did not direct the question to the staff member by not orientating his/her face to the staff member and not calling the staff member’s name. Observers were instructed to view the entire interval and to record subsequently whether or not behaviors had occurred during the interval. A plus (+) was recorded if the behavior occurred during the interval; a minus (−) was recorded if the behavior did not occur during the interval. For each child, the percentage of child-initiated questions was calculated by dividing the number of intervals with an unprompted child-initiated question by the total number of intervals, multiplied by 100.

#### Measures of Collateral Changes

In order to explore whether PRT leads to collateral changes in the children’s language skills, pragmatic skills, adaptive skills, and maladaptive behaviors, additional measures were administered during baseline and post-intervention. The CCC2 was used to measure language skills and pragmatic skills. The CCC2-NL is a 70-item questionnaire designed to measure both structural and pragmatic aspects of children’s language skills (Bishop 2003; Dutch version by; Geurts 2007). The CCC2-NL consists of ten subscales: (a) speech, (b) syntax, (c) semantics, (d) coherence, (e) inappropriate initiation, (f) stereotyped language, (g) use of context, (h) nonverbal communication, (i) social relations, and (j) interests. Based on the subscales three summary measures can be calculated: (1) general communication composite, indicating the child’s communicative competence, (2) a social-interaction deviance composite, indicating the extent of social communication difficulties versus structural language deficits, and (3) a pragmatic composite, indicating the child’s pragmatic abilities. The higher the children’s scores on these summary measures, the more impaired their skills are. In the present study, the general communication composite was used to measure language skills; the pragmatic composite was used to measure pragmatic skills. During baseline and post-intervention staff members were asked to fill in the CCC2-NL for their dyad-child. Evaluation of the psychometric qualities of the CCC2-NL demonstrated that the convergent validity, internal consistency, and test–retest reliability were sufficient and indicated that the CCC2-NL was effective in distinguishing between children with ASD, specific language impairments and attention-deficit/ hyperactivity disorder (Geurts 2007).

The Vineland-II is a standardized assessment of adaptive behavior and provides standard scores on four domains: communication, daily living skills, socialization, and motor skills (Sparrow et al. 2005). Furthermore, the Vineland-II provides an overall standard score: the adaptive behavior composite (ABC). The Vineland-II also provides a maladaptive behavior index (MBI), a composite of internalizing, externalizing and other maladaptive behaviors that may interfere with the individual’s adaptive functioning. The Vineland-II was translated into Dutch by the first author. In the present study, the ABC and the standard scores on communication, daily living skills and, socialization were used to measure adaptive skills. Higher scores indicate higher levels of adaptive functioning; lower scores indicate lower levels of adaptive functioning. The MBI was used to measure maladaptive behaviors. Higher scores indicate higher levels of maladaptive behavior; lower scores indicate lower levels of maladaptive behavior. During the last 4 weeks of baseline and first 4 weeks of post-intervention, staff members were asked to fill in the Vineland-II parent/caregiver rating form for their dyad-child.

#### Social Validity

During post-intervention, staff members were asked to fill in a questionnaire to assess the social validity of PRT in general and of the PRT staff training that was used in the present study. The questionnaire consisted of 32 statements (e.g., ‘I am willing to use PRT at my treatment group’ and ‘The individual written feedback was informative’) that were rated on a five-point Likert scale ranging from 1 (strongly disagree) to 5 (strongly agree). The questionnaire measured staff members’ attitude towards PRT and whether they considered the components of the PRT staff training as effective, relevant and pleasant.

### Inter-observer Agreement

A second observer, naïve to the purpose of the study, independently recorded 33% of the videotapes approximately evenly distributed across dyads and phases to determine inter-observer agreement for staff member-created opportunities and child-initiated questions. For opportunities, inter-observer agreement was determined using mean count-per-interval (Cooper et al. 2013). The videotapes were divided into ten 1-min intervals and a percentage of agreement between the counts of both observers was calculated for each 1-min interval. Inter-observer agreement was calculated as the average percentage of agreement across intervals. Mean overall percentage of agreement (i.e., across all videotapes) was 85% (*SD* = 12; range 50–100), indicating good inter-observer agreement (Cooper et al. 2013). For child-initiated questions, inter-observer agreement was assessed per category on an interval-by-interval basis by calculating Cohen’s kappa and prevalence-adjusted and bias-adjusted kappa (PABAK; Byrt et al. 1993; Cohen 1960). For unprompted correct child-initiated questions mean Cohen’s kappa and PABAK were 0.68 (*SD* = 0.29) and 0.86 (*SD* = 0.13), respectively. For unprompted attempts to child-initiated questions mean Cohen’s kappa and PABAK were 0.66 (*SD* = 0.24) and 0.79 (*SD* = 0.14), respectively. This indicates good to excellent inter-observer agreement (Cichetti et al. 2006; Cohen 1960).

### Data-Analysis

Data-analysis with regard to staff member-created opportunities and child-initiated questions involved visual analysis and statistical analysis. Visual analysis consisted of a systematic analysis of trend and level within and between subsequent phases for each participant, following the guidelines provided by Lane and Gast (2014). For the baseline phase, trend was calculated using the split-middle method of trend estimation. Level was analysed between subsequent phases by comparing median values.

Statistical analysis consisted of calculation of Tau_novlap_ or Tau-U (Parker et al. 2011). Tau_novlap_ and Tau-U are both effect sizes for single-case research that examine the proportion of non-overlap of data between two phases. However, Tau-U also controls for an undesirable positive baseline trend. If visual analysis indicated a strong positive baseline trend, Tau-U was calculated. Tau_novlap_ or Tau-U, the corresponding standard deviation and the *p* value were calculated for the baseline/intervention-contrast for each participant using Single Case Research (SCR), a web-based calculator for single case research analysis (Vannest et al. 2011). Tau_novlap_ or Tau-U, the corresponding standard deviation and the *p* value were calculated for non-adjacent phase contrasts (i.e., baseline/post-intervention contrast and baseline/follow-up-contrast) as well to examine change in the dependent variables during post-intervention and follow-up compared to baseline (Parker and Vannest 2012). Combined effect sizes (i.e., across staff or children) and confidence intervals were also calculated for these phase contrasts using SCR. Analyses were two-tailed and *p* value was set at 0.05. Using the guidelines of Vannest and Ninci (2015), overall effect sizes were interpreted as small (≤0.20), moderate (0.21–0.60), large (0.61–0.80), or very large (≥0.81).

Data on language skills, pragmatic skills, adaptive skills and maladaptive behaviors were analysed using the reliability of change index (RCI; Jacobson and Truax 1991) to determine whether changes in children’s questionnaire scores between baseline and post-intervention were reliable. The RCI was calculated using the following formula, where *X*_1_ en *X*_2_ represent the baseline and post-intervention scores of children, *S*_1_ the standard deviation of the sample with autism and *r*_xx_ the test–retest reliability of the used measure:

$$RCI=~\frac{{{X}_{1}}-{{X}_{2}}}{\sqrt{2({{S}_{1}}\sqrt{1-{{r}_{xx}})}}}$$

Analyses were two-tailed and *p* value was set at 0.05. Consequently, an RCI > 1.96 indicated reliable positive change; an RCI < −1.96 indicated reliable negative change (Jacobson and Truax 1991).

## Results

### Staff Member-Created Opportunities

**Fig. 1:**
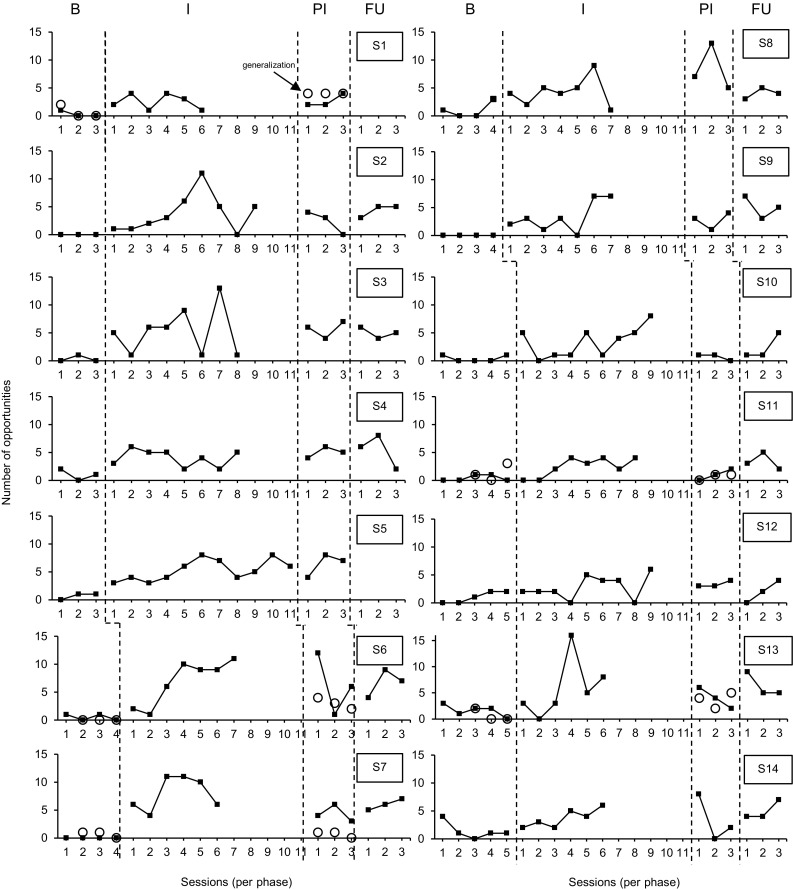
Number of opportunities during one-to-one sessions and generalization probes

Data on the number of staff member-created opportunities during one-to-one sessions are presented in Fig. 1. Visual analysis revealed a gradually increasing trend (i.e., accelerating trend line) during baseline for four staff members (S5, S8, S11, and S12), but for no staff member this positive baseline trend was statistically significant (*p* > 0.05). The median number of opportunities ranged from 0 to 2. During intervention, the median number of opportunities increased for all staff members and ranged from 2 to 9. Statistical analysis indicated that the increase in the number of opportunities was significant for 11 staff members (see Table 3). The combined Tau_novlap_ was 0.80 (90%CI 0.64–0.97; *p* < 0.001), indicating a large effect.

**Table 3:** Staff member’s values of Tau_novlap_ for one-to-one sessions

Staff	Baseline–intervention		Baseline–post-intervention		Baseline–follow-up	
	Tau_novlap_	*p*	Tau_novlap_	*p*	Tau_novlap_	*p*
1	0.89	0.039*	1.00	0.050*	–	–
2	0.89	0.027*	0.67	0.190	1.00	0.050*
3	0.86	0.032*	1.00	0.050*	1.00	0.050*
4	0.92	0.025*	1.00	0.050*	0.89	0.081
5	1.00	0.010*	1.00	0.050*	–	–
6	0.93	0.014*	0.83	0.077	1.00	0.034*
7	1.00	0.011*	1.00	0.034*	1.00	0.034*
8	0.82	0.030*	1.00	0.034*	0.92	0.052
9	0.86	0.023*	1.00	0.034*	1.00	0.034*
10	0.71	0.033*	0.27	0.551	0.73	0.101
11	0.65	0.057	0.40	0.371	1.00	0.025*
12	0.51	0.125	1.00	0.025*	0.33	0.456
13	0.63	0.082	0.73	0.101	1.00	0.025*
14	0.77	0.036*	0.27	0.551	0.87	0.052

*Significant at α = 0.05

During post-intervention, the median number of opportunities (range 1–7) increased for six staff members compared to intervention (S3, S4, S5, S8, S12, and S13), did not change for two staff members (S2 and S9), and decreased for six staff members. For all staff members the median number of opportunities remained above the baseline median. Statistical analysis revealed that, compared to baseline, eight staff members created significantly more learning opportunities during post-intervention (see Table 3). The combined Tau_novlap_ was 0.78 (90%CI 0.57–0.99; *p* < 0.001), indicating a large effect.

Follow-up sessions were conducted for 12 staff members. The median number of opportunities (range 1–8) increased for eight staff members during follow-up compared to post-intervention (S2, S4, S6, S7, S9, S11, S13, and S14), did not change for one staff member (S10), and decreased for three staff members. For all staff members the median number of opportunities remained above the baseline median. Statistical analysis demonstrated that, compared to baseline, seven staff members created significantly more learning opportunities during follow-up (see Table 3). The combined Tau_novlap_ was 0.89 (90%CI 0.67–1.11; *p* < 0.001), indicating a very large effect.

Generalization probes were conducted for five staff members during baseline and post-intervention. Data on the number of staff member-created opportunities during generalization probes are presented in Fig. 1. Visual analysis revealed an increasing trend during baseline for one staff member (S11). Although this positive baseline trend was not statistically significant (*p* > 0.05), visual analysis demonstrated a rapidly increasing baseline trend and thus Tau-U was calculated. Compared to baseline, two staff members created significantly more learning opportunities during post-intervention (see Table 4). The combined Tau across five staff members was 0.51 (90%CI 0.14–0.89; *p* = 0.02), indicating a moderate effect.

**Table 4:** Staff member’s values of Tau for generalization probes

Staff	Baseline–post-intervention	
	Tau	*p*
1	1.00^b^	0.050*
6	1.00^b^	0.050*
7	0.00^b^	1.000
11	−0.33^a^	0.513
13	0.89^b^	0.081

*Significant at α = 0.05

^a^Tau-U; ^b^Tau_novlap_

### Child-Initiated Questions

**Fig. 2:**
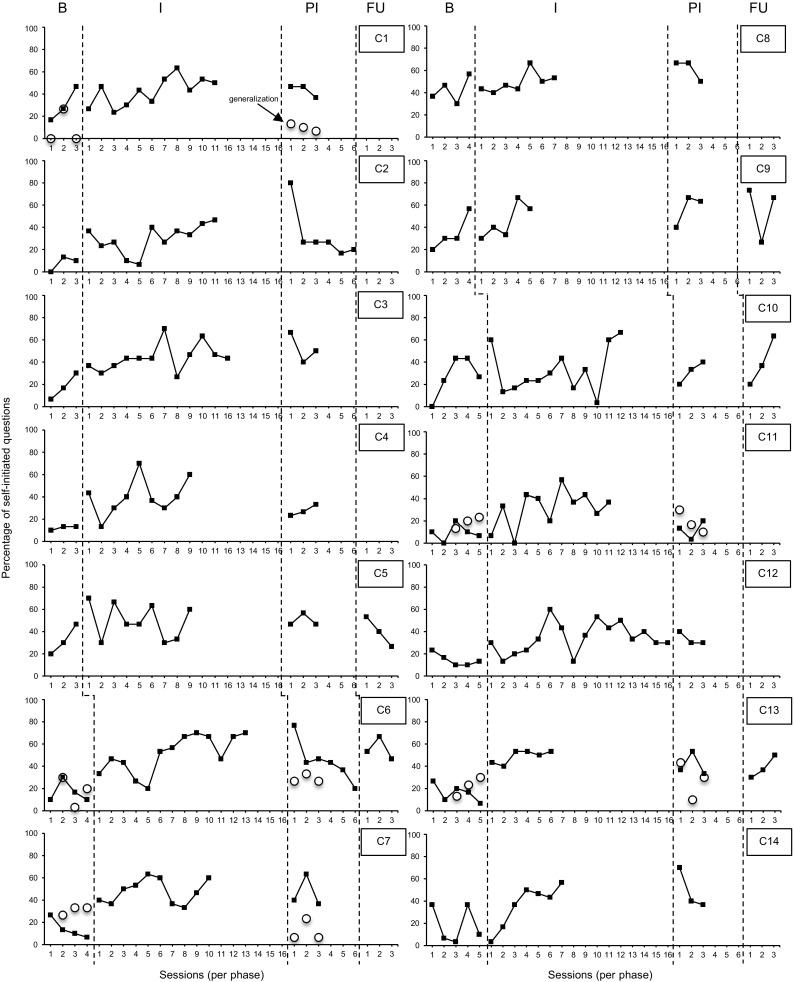
Percentage of self-initiated questions during one-to-one sessions and generalization probes

Data on the percentage of child-initiated questions during one-to-one sessions are presented in Fig. 2. Visual analysis revealed an increasing trend during baseline for 10 children (C1, C2, C3, C4, C5, C8, C9, C10, C11, and C14). Although for no child this positive baseline trend was statistically significant (*p* > 0.05), visual analysis demonstrated a rapidly increasing baseline trend for four children (C1, C3, C5, and C9) and thus Tau-U was calculated for these children. The median percentage of child-initiated questions during baseline ranged from 10.00 to 41.67. During intervention, the median percentage of child-initiated questions increased for 13 children compared to baseline and ranged from 26.67 to 53.33. Statistical analysis indicated that the increase in percentage of child-initiated questions was significant for eight children (see Table 5). The combined Tau was 0.66 (90%CI 0.50–0.82; *p* < 0.001), indicating a large effect.

**Table 5:** Children’s values of Tau for one-to-one sessions

Child	Baseline–intervention		Baseline–post-intervention		Baseline–follow-up	
	Tau	*p*	Tau	*p*	Tau	*p*
1	0.42^a^	0.389	0.22^a^	0.663	–	–
2	0.79^b^	0.043*	1.00^b^	0.020*	–	–
3	0.83^a^	0.030*	0.67^a^	0.190	–	–
4	0.93^b^	0.021*	1.00^b^	0.050*	–	–
5	0.52^a^	0.196	0.44^a^	0.383	0.00^a^	1.000
6	0.92^b^	0.007*	0.92^b^	0.019*	1.00^b^	0.034*
7	1.00^b^	0.005*	1.00^b^	0.034*	–	–
8	0.32^b^	0.395	0.83^b^	0.077	–	–
9	0.30^a^	0.462	0.42^a^	0.377	0.08^a^	0.860
10	0.07^b^	0.833	−0.07^b^	0.882	0.20^b^	0.655
11	0.69^b^	0.031*	0.27^b^	0.551	–	–
12	0.84^b^	0.006*	1.00^b^	0.025*	–	–
13	1.00^b^	0.005*	1.00^b^	0.025*	1.00^a^	0.025*
14	0.57^b^	0.104	0.87^b^	0.053	–	–

*Significant at α = 0.05

^a^Tau-U; ^b^Tau_novlap_

During post-intervention, the median percentage of child-initiated questions (range 13.33–66.67) increased for five children (C1, C3, C8, C9, and C10), did not change for one child (C5), and decreased for eight children, but remained above the baseline median for all children. Statistical analysis demonstrated that, compared to baseline, six children initiated significantly more questions during post-intervention (see Table 5). The combined Tau was 0.69 (90%CI 0.48–0.89; *p* < 0.001), indicating a large effect.

Follow-up sessions were conducted for five children. The median percentage of child-initiated questions (range 36.67–66.67) increased for three children during follow-up compared to post-intervention (C6, C9, and C10), did not change for one child (C13), and decreased for one child. For all children the median percentage of child-initiated questions remained above the baseline median. Statistical analysis indicated that, compared to baseline, two children initiated significantly more questions during follow-up (see Table 5). The combined Tau was 0.47 (90%CI 0.12–0.81; *p* = 0.03), indicating a moderate effect.

Generalization probes were conducted for five children during baseline and post-intervention. Data for the percentage of child-initiated questions during generalization probes are presented in Fig. 2. Visual analysis demonstrated an increasing trend during baseline for three children (C7, C11, and C13). Although this positive baseline trend was not statistically significant (*p* > 0.05), visual analysis indicated a rapidly increasing baseline trend for two children (C11 and C13) and thus Tau-U was calculated for these children. Statistical analysis revealed that the percentage of child-initiated questions decreased significantly for one child (see Table 6). For the other children, the percentage of child-initiated questions did not change significantly during generalization probes. The combined Tau was −0.20 (90%CI 0.59–0.18; *p* = 0.39), indicating a small negative effect.

**Table 6:** Children’s values of Tau for generalization probes

Child	Baseline–post-intervention	
	Tau	*p*
1	0.33^a^	0.513
6	0.56^a^	0.275
7	−1.00^a^	0.050*
11	−0.44^b^	0.383
13	−0.11^b^	0.827

*Significant at α = 0.05

^a^Tau_novlap_; ^b^Tau-U

### Collateral Improvements

Table 7 presents data on language, pragmatic, and adaptive skills and maladaptive behaviors as measured with the CCC2-NL and Vineland-II, and the number of children that demonstrated reliable change in the these behaviors between baseline and post-intervention. Although mean scores on the general communication composite and pragmatic composite of the CCC2-NL changed in the expected direction, for none of the children changes in these scores were reliable. For three children (C2, C5, and C8) improvements in the adaptive behavior composite were reliable. For one of these children (C8) the RCI indicated a reliable improvement in the subdomain of daily living skills. One child (C13) demonstrated a reliable improvement in the subdomain of socialization. Of these four children, child 2 and child 13 also demonstrated significant improvements in child-initiated questions. For one child (C6), the RCI indicated a reliable decrease in the overall level of adaptive skills and, more specifically in the subdomains of daily living skills and socialization, despite significant improvements in child-initiated questions. Three children (C6, C13, and C14) demonstrated reliable reductions in maladaptive behaviors. For two children (C6 and C13) this reduction accompanied a significant increase in child-initiated questions.

**Table 7:** Descriptive statistics and frequencies of positive reliable change for collateral improvement

Measure	Baseline		Post-intervention		Positive reliable change	
	Mean	SD	Mean	SD	N	%
CCC2-NL: general communication composite	111.93	13.83	105.71	15.77	0	0
CCC2-NL: pragmatic composite	58.21	6.53	54.93	7.12	0	0
Vineland-II: adaptive behavior composite	73.79	8.79	77.50	9.20	3	21
Vineland-II: communication	76.14	7.29	78.57	8.65	0	0
Vineland-II: daily living skills	81.14	15.69	84.14	14.44	1	7
Vineland-II: socialization	70.21	8.29	75.93	9.39	1	7
Vineland-II: maladaptive behavior composite	19.43	1.34	18.71	1.20	3	21

### Social Validity

Overall staff members rated the PRT staff training as highly effective (*M* = 4.6), highly relevant (*M* = 4.5), and highly satisfactory (*M* = 4.2). With regard to the components of the training, video feedback and written feedback were rated as most effective with mean scores of 4.8 and 4.7, respectively. The role-plays were rated as least effective (*M* = 3.7). Although staff members rated practicing the PRT-techniques during one-to-one PRT-sessions as highly effective (*M* = 4.4), their rating of the opportunities to practice between the training days was less positive (*M* = 3.3). Staff members’ attitudes towards PRT were positive at post-training (*M* = 4.3). Moreover, staff members indicated to implement PRT as much as possible at the inpatient treatment facility (*M* = 4).

## Discussion

In the present study, staff members of an inpatient treatment facility in the Netherlands for school-aged children with ASD were taught to create opportunities for question-asking through staff training in PRT. Eleven of the 14 staff members created significantly more opportunities during intervention, indicating that staff training in PRT is effective for this purpose. However, generalization of creating opportunities to group situations was limited. Post-intervention and follow-up data demonstrated that most staff members maintained their skills over time. Furthermore, 8 of the 14 children initiated significantly more questions as a result of intervention. However, only a minority of the children maintained these skills over time. Generalization of child-initiated questions to group situations and collateral changes in language, pragmatic and adaptive skills and maladaptive behaviors did not occur.

The present study confirms findings of Huskens et al. (2012) indicating that staff can be taught to create opportunities for question-asking using PRT. Furthermore, this study adds to the growing evidence base supporting the use of PRT to improve question-asking in school-aged children with ASD (e.g., Dogget et al. 2013; Huskens et al. 2012; Robinson 2011). Until now, studies targeting question-asking focused on the acquisition of questions within only one communicative function (e.g., Betz et al. 2010; Dogget et al. 2013). The present study extends these studies by showing that children with ASD can acquire multiple questions with various communicative functions in the context of natural daily activities.

Both staff members and children with ASD did not generalize the targeted skills to group situations. Research on implementation of PRT in group situations is limited, but studies in school settings have indicated that PRT techniques need to be adapted for implementation in classrooms and that teachers required additional training to be able to implement PRT in group settings with multiple children (Stahmer et al. 2012), suggesting that staff members also may require additional skills and training to create opportunities and implement PRT in group situations. Because of limited generalization of staff members’ skills it is not surprising that children’s question-asking skills did not improve in group situations. This suggests that children relied on staff members’ cues and prompts to initiate questions in these situations. Self-management might be helpful to promote generalization of question-asking to situations where staff members’ cues are less frequent or absent (e.g., Koegel et al. 2014).

Although the number of opportunities increased for most staff members, there remained a great deal of variability in responding between staff. Staff characteristics may account for this variability (Durlak and DuPre 2008; Peters-Scheffer et al. 2013; Symes et al. 2005). For example, Peters-Scheffer et al. (2013) examined the relationship between procedural fidelity of DTT and therapist personality traits, attitude towards individuals with disabilities, and therapist-child relationship. Results indicated that procedural fidelity was significantly related to these staff characteristics. The procedural fidelity of PRT might also be associated with these and other staff characteristics. Because the sample size of the present study was too small to explore the association between procedural fidelity of PRT staff characteristics, future research should address this topic.

Similarly, intervention outcomes across children were also highly variable. This outcome variability is consistent with the results of a systematic review on PRT (Verschuur et al. 2014) and evaluations of ABA interventions (e.g., Peters-Scheffer et al. 2011; Reichow 2012; Vivanti et al. 2014). Behavioral intervention outcomes are associated with child characteristics, for example age, language proficiency, pre-intervention cognitive skills, and autism severity (e.g., Ben-Itzchak and Zachor 2011; Perry et al. 2013; Smith et al. 2015). However, these characteristics do not seem to explain variability in children’s question-asking skills in the present study, because these characteristics also varied across children who did not benefit from PRT. Future research should investigate whether these and other child characteristics (e.g., psychiatric comorbidity and maladaptive behaviors) are associated with outcomes of PRT for school-aged children with ASD. In addition to variability across children, question-asking also varied across intervention sessions within individual children. This suggests that, although children might have acquired the skills to initiate questions, they are not yet able to use these skills consistently. Factors that could explain this variable performance within children are currently unknown.

Whereas other studies reported generalized improvements as a result of PRT (e.g., Baker-Ericzén et al. 2007; Mohammadzaheri et al. 2014, 2015), the present study did not find significant (i.e., reliable) collateral changes in children’s language, pragmatic, and adaptive skills and maladaptive behaviours, despite the fact that identical measures were used (i.e., CCC2 and Vineland-II). Different methods of data-analysis may account for these inconsistent results. Other studies analysed changes in mean scores across children, for example using paired-sample *t*-tests. The present study analysed changes in collateral skills using the RCI, which represents individual changes and takes measurement errors into account (Jacobson and Truax 1991). Exploratory paired-sample *t*-tests and Wilcoxon signed-rank tests were conducted to compare results across analyses and demonstrated statistically significant improvements in children’s language, pragmatic, and overall adaptive skills. This comparison suggests that although mean scores across children might have changed significantly, these changes were smaller than the questionnaires’ standard errors of measurement and thus not reliable according to an RCI approach. Future studies investigating generalized improvements as a result of PRT should take measurement errors into account by analysing data at the individual level.

There are several limitations to the present study. First, the number of staff member-created opportunities is presumably underestimated, because only opportunities that resulted in self-initiated questions were considered correct to take the child’s motivation into account. Motivation is often defined as children’s responsiveness to social and environmental stimuli (Koegel et al. 2001). If staff gained the child’s attention, but the child did not ask a question, it was assumed that staff did not follow the child’s motivation and no opportunity was scored. However, this could have led to an underestimation of the number opportunities. Second, all questions were coded as self-initiated questions and no distinction was made between self-initiated questions with different communicative functions, although social questions (e.g., ‘How was your weekend?’*)* have more potential to improve children’s social success than functional questions (e.g., ‘Can I have the blocks?’). Third, baseline trend was positive for ten children. This suggests that children’s question-asking skills might improve without PRT, but it could also be possible that staff members unintentionally or naturally implemented some antecedent or consequent PRT techniques during baseline, for example by responding to children’s spontaneous questions (e.g., Raulston et al. 2013). Fourth, due to high level of attrition follow-up sessions were conducted for only five children. Results concerning maintenance of question-asking skills should thus be interpreted with caution. Fifth, because the researcher collected generalization probes, reactive effects could have occurred during these probes (Cooper et al. 2013). Similarly, increases in staff member-created opportunities during baseline, post-intervention, or follow-up could be a result of increased monitoring, because staff members were instructed to record these sessions and were thus aware of being observed. Finally, collateral skills were measured using questionnaires. In order to gain more objective data, however, direct assessment methods such as observation can be considered more suitable to measure behavior change (Cooper et al. 2013).

Despite these limitations, the results of this study are promising as they indicate that PRT staff training is effective in teaching inpatient staff to create opportunities for question-asking. Moreover, question-asking skills of some school-aged children with ASD improved as a result of PRT. Further research is necessary to investigate training procedures that promote generalized, consistent, and continuous implementation of PRT by staff across situations and to identify staff and child characteristics associated with fidelity of PRT implementation respectively PRT outcomes.

## References

[CR64] American Psychiatric Association (2000). Diagnostic and statistical manual of mental disorders IV-TR.

[CR1] American Psychiatric Association (2013). Diagnostic and statistical manual of mental disorders.

[CR2] Baker-Ericzén MJ, Stahmer AC, Burns A (2007). Child demographics associated with outcomes in a community-based pivotal response training program. Journal of Positive Behavior Interventions.

[CR3] Ben-Itzchak E, Zachor DA (2011). Who benefits from early intervention in autism spectrum disorders?. Research in Autism Spectrum Disorders.

[CR4] Betz AM, Higbee TS, Pollard JS (2010). Promoting generalization of mands for information used by young children with autism. Research in Autism Spectrum Disorders.

[CR5] Bishop DMV (2003). The children’s communication checklist-2.

[CR6] Byrt T, Bishop J, Carlin JB (1993). Bias, prevalence and kappa. Journal of Clinical Epidemiology.

[CR7] Charlop MH, Milstein JP (1989). Teaching autistic children conversational speech using video modeling. Journal of Applied Behavior Analysis.

[CR8] Cicchetti D, Bronen R, Spencer S, Haut S, Berg A, Oliver P, Tyrer P (2006). Rating scales, scales of measurement, issues of reliability. Resolving some critical issues for clinicians and researchers. The Journal of Nervous and Mental Disease.

[CR9] Cidav Z, Lawer L, Marcus SC, Mandell DS (2013). Age-related variation in health service use and associated expenditures among children with autism. Journal of Autism and Developmental Disorders.

[CR10] Cohen J (1960). A coefficient of agreement for nominal scales. Educational and Psychological Measurement.

[CR11] Cooper JO, Heron TE, Heward WL (2013). Applied behavior analysis.

[CR12] De Bildt A, Greaves-Lord K, de Jonge M (2013). ADOS-2, autisme diagnostisch observatie schema.

[CR13] Dogget RA, Krasno AM, Koegel LK, Koegel RL (2013). Acquistion of multiple questions in the context of social conversation in children with autism. Journal of Autism and Developmental Disorders.

[CR14] Dotto-Fojut KM, Reeve KF, Townsend DB, Progar PR (2011). Teaching adolescents with autism to describe a problem and request assistance during simulated vocational tasks. Research in Autism Spectrum Disorders.

[CR15] Durlak JA, DuPre EP (2008). Implementation matters: A review of research on the influence of implementation on program outcomes and the factors affecting implementation. American Journal of Community Psychology.

[CR16] Geurts HM (2007). CCC2-NL: Children’s communication checklist-2.

[CR17] Hauck M, Fein D, Waterhouse L, Feinstein C (1995). Social initiations by autistic children to adults and other children. Journal of Autism and Developmental Disorders.

[CR18] Hudry K, Aldred C, Wigham S, Green J, Leadbitter K, Temple K, PACT Consortium (2013). Predictors of parent–child interaction style in dyads with autism. Research in Developmental Disabilities.

[CR19] Huskens B, Reijers H, Didden R (2012). Staff training effective in increasing learning opportunities for school-aged children with autism spectrum disorders. Developmental Neurorehabilitation.

[CR20] Ingvarsson ET, Hollobaugh T (2010). Acquisition of intraverbal behavior: Teaching children with autism to mand for answers to questions. Journal of Applied Behavior Analysis.

[CR21] Jacobson NS, Truax P (1991). Clinical significance: A statistical approach to defining meaningful change in psychotherapy research. Journal of Consulting and Clinical Psychology.

[CR22] Kazdin AE (2011). Single-case research designs: Methods for clinical and applied settings.

[CR23] Koegel LK, Carter CM, Koegel RL (2003). Teaching children with autism self- initiations as a pivotal response. Topics in Language Disorders.

[CR24] Koegel LK, Koegel RL, Green-Hopkins I, Barnes CC (2010). Brief report: Question-asking and collateral language acquisition in children with autism. Journal of Autism and Developmental Disorders.

[CR25] Koegel LK, Koegel RL, Shoshan Y, McNerney EK (1999). Pivotal response intervention II: Preliminary long-term outcome data. Journal of the Association for Persons with Severe Handicaps.

[CR26] Koegel LK, Park MN, Koegel RL (2014). Using self-management to improve the reciprocal social conversation of children with autism spectrum disorder. Journal of Autism and Developmental Disorders.

[CR27] Koegel LK, Vernon TW, Koegel RL, Koegel BL, Paullin AW (2012). Improving social engagement and initiations between children with autism spectrum disorder and their peers in inclusive settings. Journal of Positive Behavior Interventions.

[CR28] Koegel RL, Bradshaw JL, Ashbaugh K, Koegel LK (2014). Improving question-asking initiations in young children with autism using pivotal response treatment. Journal of Autism and Developmental Disorders.

[CR29] Koegel RL, Koegel LK (2006). Pivotal response treatments for autism: Communication, social, and academic development.

[CR30] Koegel RL, Koegel LK, McNerney EK (2001). Pivotal areas in intervention for autism. Journal of Clinical Child & Adolescent Psychology.

[CR31] Kort W, Schittekatte M, Dekker PH, Verhaeghe P, Compaan EL, Bosmans M (2005). *WISC-III*^*NL*^. *Handleiding en verantwoording. Nederlandse bewerking*.

[CR32] Lane JD, Gast DL (2014). Visual analysis in single case experimental design studies: Brief review and guidelines. Neuropsychological Rehabilitation.

[CR33] Lord C, Rutter M, DiLavore PC, Risi S, Gotham K, Bishop SL (2012). Autism diagnostic observation schedule.

[CR34] MacDuff GS, Krantz PJ, McClannahan LE, Maurice C, Green G, Foxx RM (2001). Prompts and prompt-fading strategies for people with autism. Making a difference: Behavioral intervention for autism.

[CR35] Mandell DS (2008). Psychiatric hospitalization among children with autism spectrum disorders. Journal of Autism and Developmental Disorders.

[CR36] Mohammadzaheri F, Koegel LK, Rezaee M, Bakhshi E (2015). A randomized clinical trial comparison between pivotal response treatment (PRT) and adult-driven applied behavior analysis (ABA) intervention on disruptive behaviors in public school children with autism. Journal of Autism and Developmental Disorders.

[CR37] Mohammadzaheri F, Koegel LK, Rezaee M, Rafiee SM (2014). A randomized clinical trial comparison between pivotal response treatment (PRT) and structured applied behavior analysis (ABA) intervention for children with autism. Journal of Autism and Developmental Disorders.

[CR38] Palmen A, Didden R, Arts M (2008). Improving question asking in high-functioning adolescents with autism spectrum disorders: Effectiveness of small-group training. Autism: The International Journal of Research and Practice.

[CR39] Parker RI, Vannest, K J (2012). Bottom-up analysis of single-case research designs. Journal of Behavioral Education.

[CR40] Parker RI, Vannest KJ, Davis JL (2010). Effect size in single case research: A review of nine non-overlap techniques. Behavior Modification.

[CR41] Perry A, Blacklock K, Dunn Geier J (2013). The relative importance of age and IQ as predictors of outcomes in intensive behavioral intervention. Research in Autism Spectrum Disorders.

[CR42] Peters-Scheffer N, Didden, Korzilius H, Sturmey P (2011). A meta-analytic study on the effectiveness of comprehensive ABA-based early intervention programs for children with Autism Spectrum Disorders. Research in Autism Spectrum Disorders.

[CR43] Peters-Scheffer N, Didden, Korzilius H, Sturmey P (2013). Therapist characteristics predict discrete trial teaching procedural fidelity. Intellectual and Developmental Disabilities.

[CR44] Raulston T, Carnett A, Lang R, Tostanoski A, Lee A, Machalicek W, Lancioni GE (2013). Teaching individuals with autism spectrum disorder to ask questions: A systematic review. Research in Autism Spectrum Disorders.

[CR45] Reichow B (2012). Overview of meta-analyses on early intensive be- havioral intervention for young children with autism spectrum dis-order. Journal of Autism and Developmental Disorders.

[CR46] Reid DH, Fitch, W H, Matson JL, Sturmey P (2011). Training staff and parents: Evidence-based approaches. International handbook of autism and pervasive developmental disorders.

[CR47] Robinson SE (2011). Teaching paraprofessional of students with autism to implement pivotal response treatment in inclusive school settings using a brief video feedback training package. Focus on Autism and Other Developmental Disabilities.

[CR48] Rutter M, Bailey A, Lord C (2003). Social communication questionnaire.

[CR49] Sherer MR, Schreibman L (2005). Individual behavioral profiles and predictors of treatment effectiveness for children with autism. Journal of Clinical and Consulting Psychology.

[CR50] Siegel M, Gabriels RL (2014). Psychiatric hospital treatment of children with autism and serious behavioral disturbance. Child and Adolescent Psychiatric Clinics of North America.

[CR51] Smith IM, Flanagan, H E, Garon N, Bryson SE (2015). Effectiveness of community-based early intervention based on pivotal response treatment. Journal of Autism and Developmental Disorders.

[CR52] Sparrow SS, Cicchetti DV, Balla DA (2005). Vineland adaptive behavior scales.

[CR53] Stahmer AC, Suhrheinrich J, Reed S, Schreibman L (2012). What works for you? Using teacher feedback to inform adaptations of pivotal response training for classroom use. Autism Research and Treatment.

[CR54] Stone WL, Caro-Martinez, L M (1990). Naturalistic observations of spontaneous communication in autistic children. Journal of Autism and Developmental Disorders.

[CR55] Stone WL, Ousley, O Y, Yoder, P J, Hogan KL, Hepburn SL (1997). Nonverbal communication in 2-and 3-year-old children with autism. Journal of Autism and Developmental Disorders.

[CR56] Symes MD, Remington B, Brown T, Hastings RP (2006). Early intensive behavioral intervention for children with autism: Therapists’ perspectives on achieving procedural fidelity. Research in Developmental Disabilities.

[CR57] van Dijk H, Tellegen PJ (2004). NIO, Nederlandse Intelligentietest voor Onderwijsniveau. Handleiding en Verantwoording.

[CR58] Vannest KJ, Ninci J (2015). Evaluation intervention effects in single-case research designs. Journal of Counseling & Development.

[CR59] Vannest, K. J., Parker, R. I., & Gonen, O. (2011). Single case research: Web-based calculators for SCR analysis. (Version 1.0) [Web-based application]. College Station, TX: Texas A&M University. Retrieved January 12, 2016, from http://singlecaseresearch.org.

[CR60] Verschuur R, Didden R, Lang R, Sigafoos J, Huskens B (2014). Pivotal response treatment for children with autism spectrum disorders: A systematic review. Review Journal of Autism and Developmental Disorders.

[CR61] Vivanti G, Prior M, Williams K, Dissanayake C (2007). Predictors of outcomes in autism early intervention: Why don’t we know more?. New Treatment Perspectives in Autism Spectrum Disorders.

[CR62] Wert BY, Neisworth JT (2003). Effects of video self-modeling on spontaneous requesting in children with autism. Journal of Positive Behavior Interventions.

[CR63] Wetherby AM, Prutting CA (1984). Profiles of communicative and cognitive-social abilities in autistic children. Journal of Speech, Language, and Hearing Research.

